# Bayesian sequential approach to monitor COVID-19 variants through test positivity rate from wastewater

**DOI:** 10.1128/msystems.00018-23

**Published:** 2023-07-25

**Authors:** J. Cricelio Montesinos-López, Maria L. Daza-Torres, Yury E. García, César Herrera, C. Winston Bess, Heather N. Bischel, Miriam Nuño

**Affiliations:** 1 Department of Public Health Sciences, University of California Davis, Davis, California, USA; 2 Department of Mathematics, Purdue University, West Lafayette, Indiana, USA; 3 Department of Civil and Environmental Engineering, University of California Davis, Davis, California, USA; Princeton University, Princeton, New Jersey, USA

**Keywords:** SARS-CoV-2, COVID-19, test positivity rate (TPR), wastewater-based epidemiology (WBE), effective reproductive number, Bayesian sequential data assimilation

## Abstract

Deployment of clinical testing on a massive scale was an essential control measure for curtailing the burden of severe acute respiratory syndrome coronavirus 2 (SARS-CoV-2) infections and the magnitude of the COVID-19 (coronavirus disease 2019) pandemic during its waves. As the pandemic progressed, new preventive and surveillance mechanisms emerged. Implementation of vaccine programs, wastewater (WW) surveillance, and at-home COVID-19 antigen tests reduced the demand for mass SARS-CoV-2 testing. Unfortunately, reductions in testing and test reporting rates also reduced the availability of public health data to support decision-making. This paper proposes a sequential Bayesian approach to estimate the COVID-19 test positivity rate (TPR) using SARS-CoV-2 RNA concentrations measured in WW through an adaptive scheme incorporating changes in virus dynamics. The proposed modeling framework was applied to WW surveillance data from two WW treatment plants in California; the City of Davis and the University of California, Davis campus. TPR estimates are used to compute thresholds for WW data using the Centers for Disease Control and Prevention thresholds for low (<5% TPR), moderate (5%–8% TPR), substantial (8%–10% TPR), and high (>10% TPR) transmission. The effective reproductive number estimates are calculated using TPR estimates from the WW data. This approach provides insights into the dynamics of the virus evolution and an analytical framework that combines different data sources to continue monitoring COVID-19 trends. These results can provide public health guidance to reduce the burden of future outbreaks as new variants continue to emerge.

**IMPORTANCE:**

We propose a statistical model to correlate WW with TPR to monitor COVID-19 trends and to help overcome the limitations of relying only on clinical case detection. We pose an adaptive scheme to model the nonautonomous nature of the prolonged COVID-19 pandemic. The TPR is modeled through a Bayesian sequential approach with a beta regression model using SARS-CoV-2 RNA concentrations measured in WW as a covariable. The resulting model allows us to compute TPR based on WW measurements and incorporates changes in viral transmission dynamics through an adaptive scheme.

## INTRODUCTION

Effectively monitoring the evolution of the COVID-19 (coronavirus disease 2019) pandemic and controlling the spread of disease remains a major public health challenge. Statistical and mathematical models are important components of effective monitoring systems to track COVID-19 cases, hospitalizations, and deaths (1, 2). Unfortunately, the rapid evolution of severe acute respiratory syndrome coronavirus 2 (SARS-CoV-2) and variable community responses to public health interventions complicate the development of robust mathematical models. Classic models in epidemiology are limited in their ability to capture the complex dynamics of the evolving pandemic (3, 4). Data availability and quality have also changed over time (5, 6). Deployment of clinical testing on a massive scale (“mass testing”) was an essential control measure for curtailing the burden of COVID-19, particularly during its early phases. As the pandemic progressed, new interventions and monitoring strategies surged, including vaccine programs, wastewater (WW) surveillance (7, 8), and at-home COVID-19 antigen tests (9). In this study, we recognize the increasingly important role of WW surveillance data in disease monitoring and use WW data and statistical modeling to infer public health metrics.

Mass testing, contact tracing, isolation, and mobility restrictions made it possible to estimate the burden of disease during the early phases of the pandemic (10, 11). The return to “normal” was accompanied by a decrease in COVID-19 clinical testing programs and an increase in at-home diagnostic tests. These changes diminished the utility of individual case data for public health decision-making. Using the number of confirmed cases to determine the prevalence of disease in a community introduces bias since case counts depend on the volume of tests conducted, testing priorities, and the timing of case detection (12, 13). Test positivity rate (TPR) has been shown to be a better indicator of disease spread than confirmed cases because it considers both tests conducted and cases detected (14, 15).

During the COVID-19 pandemic, public health officials commonly used the TPR to infer the adequacy of population-level testing and the extent of SARS-CoV-2 transmission in a population (15 - 17). A low TPR indicates a low level of virus transmission and reflects a high surveillance capacity and rapid case identification. In contrast, a higher TPR indicates a higher level of virus transmission but also suggests that too few tests are being conducted and many infected individuals are likely undetected (18 - 20).

At the beginning of the pandemic, the World Health Organization (WHO) recommended a TPR threshold of 5% to declare COVID-19 transmission under control (21). As the number of people being tested for COVID-19 declined over time, the TPR alone was deemed insufficient to assess community-level transmission. Limited levels of testing meant that public health authorities focused on passive case-finding (i.e., only those considered most likely to be infected due to symptoms or contacts were tested). As a result, TPR tended to be artificially high, and models using TPR as input tended to overestimate the proportion of people infected. This is contrary to models based only on observed cases, which may underestimate COVID-19 prevalence (22). Despite these limitations, TPR can still provide a reasonable estimate of the extent of an outbreak if TPR is combined with additional information.

Public health authorities are turning to wastewater-based epidemiology (WBE) as an alternative strategy for less-biased population-level surveillance of SARS-CoV-2 RNA. WBE uses biomarkers in WW to monitor trends in community-level health indices. WBE methods have been used to detect changes in drug consumption (23), dietary patterns (24), and the circulation of pathogens like poliovirus and norovirus (25). SARS-CoV-2 RNA measurements in WW can be used to understand COVID-19 epidemiology because infected individuals shed the virus into the sewer system throughout their infection (8). During the COVID-19 pandemic, SARS-CoV-2 RNA concentrations in WW were shown to correlate strongly with confirmed cases in numerous studies (e.g., 8, 22, 26). However, some studies have shown that the relationship between WW and COVID-19 clinical cases varies over time (27, 28). This relationship is affected by many factors, including testing availability and practices, public health policies, social behaviors, vaccine uptake, acquired immunity, and the emergence of new variants that may impact fecal shedding (27, 29, 30). Xiao et al. (27) attributed changes in the ratio of WW signal to daily new positive clinical tests to insufficient testing in the general population (i.e., inadequate testing to capture the exponential growth of actual COVID-19 cases).

In this paper, we correlate WW with TPR to monitor COVID-19 trends using high-quality clinical testing and WW data from Davis, California. We pose an adaptive scheme to model the nonautonomous nature of the prolonged COVID-19 pandemic. The TPR is modeled through a sequential Bayesian approach (3) with a beta regression using the WW viral loads as a covariable. The resulting model allows us to compute a TPR based on WW measurements and incorporates changes in viral transmission dynamics through an adaptive scheme. The TPR estimate is used to calculate values for WW data that correspond to TPR thresholds using criteria proposed by the U.S. Centers for Disease Control and Prevention (CDC) in 2021 (31). The TPR thresholds indicated low transmission for a TPR <0.05, moderate transmission for a TPR within a range of 0.05 and 0.08, substantial transmission for a TPR within a range of 0.08 and 0.1, and high transmission for a TPR ≥0.1. Due to uncertainties in relating WW data to COVID-19 case counts, public health authorities typically evaluate trends in WW data to assess changes in infection rates rather than absolute thresholds. It may also be helpful for public health authorities to interpret WW data in terms of TPR. Our modeling approach provides insights into the evolution of virus transmission dynamics and a methodology that combines different sources of information to continue monitoring COVID-19 trends.

## RESULTS

**Fig 1:**
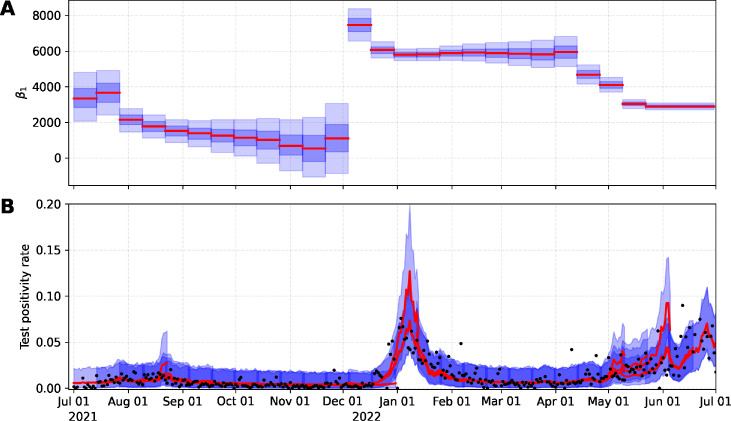
Posterior distribution of $\beta_{1}$ (**A**) and estimated TPR for the City of Davis (**B**). Red-solid line and blue-shadow area describe the median and 95% prediction intervals, respectively.

**Fig 2:**
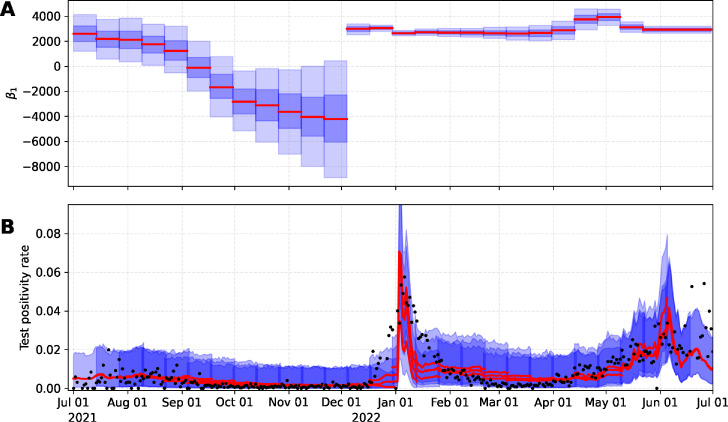
Posterior distribution of $\beta_{1}$ (**A**) and estimated TPR for UC Davis (**B**). Red-solid line and blue-shadow area describe the median and 95% prediction intervals, respectively.

The analytical framework was developed using data from the City of Davis (Davis) and replicated for the University of California, Davis campus (UC Davis). To display changes in the relation between TPR and WW signal across variants, we estimate the TPR using the proposed sequential model and compute the posterior distribution for the parameter $\beta_{1}$ over time (Fig. 1 and 2, top-panel). The results show how the relation of TPR and WW signal changed over time, mainly as new variants emerged. Note that while these were new periods of a variant emerging, this also likely corresponded to a period of under-testing when variants began to surge.

**Fig 3:**
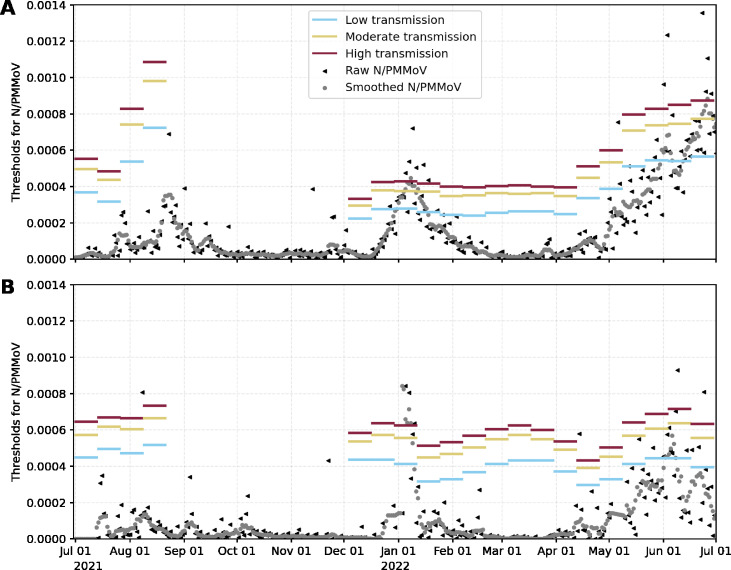
Wastewater thresholds over time for (**A**) the City of Davis and (**B**) UC Davis. Blue, yellow, and red horizontal lines correspond to low, moderate, and high transmission thresholds, respectively. Raw (black triangles) and smoothed *N*/PMMoV WW data (gray dots).

The Algorithm 1 was used to compute the thresholds for WW data with the estimated TPR in each time window. We did not calculate thresholds for the period of September through December 2021 since the corresponding posterior distribution for $\beta_{1}$ contained zero. In other words, the probability that the $\beta_{1}$ parameter will be zero was positive, suggesting that there is no significant association between WW data and TPR (Fig. 1 and 2). We illustrate the estimated thresholds for WW corresponding to low, moderate, substantial, and high transmission thresholds proposed by the CDC (Fig. 3). High variability in the threshold estimation coincides with the emergence of new variants. This variability is reduced in the period where the variant is dominant. WW thresholds were determined by calculating the mean of the estimated thresholds in each period; see Table 1. Thresholds are reported as *N*/PMMoV (*N* gene copies per gram dry weight solids normalized by mild pepper mottle virus gene copies per gram dry weight solids).

**TABLE 1:** WW thresholds were determined by calculating the mean of the estimated thresholds in each period

	Wastewater thresholds		
	Delta	Omicron	BA.2–5
**Davis**			
Low	< 5.72 × 10^−4^	< 2.61 × 10^−4^	< 4.82 × 10^−4^
Moderate	(5.72 × 10^−4^, 7.76 × 10^−4^)	(2.61 × 10^−4^, 3.65 × 10^−4^)	(4.82 × 10^−4^, 6.58 × 10^−4^)
Substantial	(7.76 × 10^−4^, 8.63 × 10^−4^)	(3.65 × 10^−4^, 4.10 × 10^−4^)	(6.58 × 10^−4^, 7.44 × 10^−4^)
High	> 8.63 × 10^−4^	> 4.10 × 10^−4^	> 7.44 × 10^−4^
**UC Davis**			
Low	< 5.16 × 10^−4^	< 3.93 × 10^−4^	< 3.87 × 10^−4^
Moderate	(5.16 × 10^−4^, 6.53 × 10^−4^)	(3.93 × 10^−4^, 5.28 × 10^−4^)	(3.87 × 10^−4^, 5.36 × 10^−4^)
Substantial	(6.53 × 10^−4^, 7.17 × 10^−4^)	(5.28 × 10^−4^, 5.89 × 10^−4^)	(5.36 × 10^−4^, 6.03 × 10^−4^)
High	> 7.17 × 10^−4^	> 5.89 × 10^−4^	> 6.03 × 10^−4^

**Fig 4:**
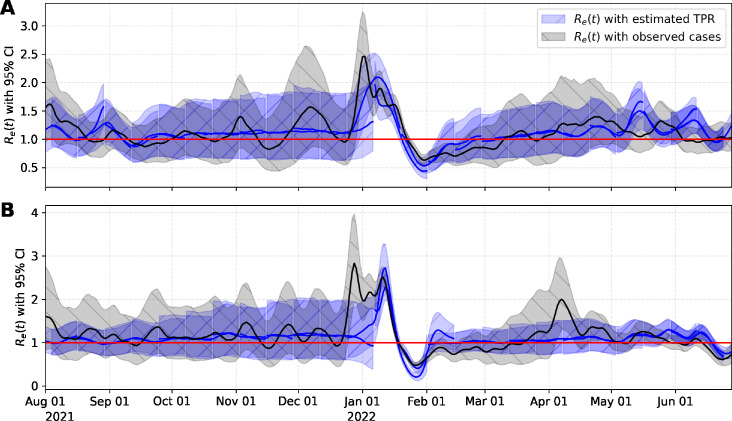
Effective $R_{e}$ of (**A**) the City of Davis and (**B**) UC Davis computed with observed cases (black line and gray shadow) and with the median of the predicted TPR multiplied by the average number of tests performed in the study period (blue lines and blue shadow). Solid lines and shaded regions illustrate the median and 95% prediction intervals, respectively.

Fig. 4 illustrates the effective reproductive number ( $R_{e}$ ), which was computed assuming the median of the predicted TPR multiplied by the average number of tests, N , performed per day for Davis ( N=1,198 ) and UC Davis ( N=2,381 ), in the study period. We also compare $R_{e}$ computed with observed cases. $R_{e}$ trends determined from WW were similar in magnitude and depicted similar trends for $R_{e}$ calculated using observed cases during the periods analyzed.

## DISCUSSION

Substantial changes in test availability and test-seeking behavior may confound COVID-19 case count estimates in a community (22, 32). The TPR may provide a more accurate reflection of the state of the epidemic (15). TPR is an important metric because it indicates how widespread an outbreak is within a particular area where testing is being conducted and whether current levels of testing are sufficient to accurately capture levels of disease transmission (16, 18). Increments in TPR can indicate that it may be a good time to incorporate restrictions to slow the spread of disease (18).

This study proposes a sequential Bayesian framework to model the COVID-19 TPR via WW data for near real-time monitoring of the COVID-19 pandemic. The TPR is modeled as a reparametrized beta regression, and the parameters are estimated using a Bayesian approach. The changing dynamics of the virus and data availability impose challenges in the development of actionable mathematical models for the surveillance and monitoring of COVID-19 (3, 33). Here, we propose an adaptive modeling framework as an alternative to overcome some of the limitations of traditional models. The adaptive capacity of the model is well suited to capture the variability in virus trends over time by leveraging knowledge gained.

We then use the model developed to offer a retrospective estimate of WW thresholds (for the settled solids analytical method performed) that corresponded to TPR thresholds recommended by the CDC, for community transmission levels of SARS-CoV-2. The WW thresholds determined for UC Davis appeared more stable through time and through waves of different SARS-CoV-2 variants than WW thresholds estimated for the City of Davis. This can be explained by the fact that the UC Davis TPR was nearly always less than 5% while the City of Davis TPR exceeded 5% periodically over the study period. Confidence in disease dynamics breaks down as TPR rises above 5% (21). The WW thresholds estimated for the City of Davis were similar to those for UC Davis when TPR remained low but increased dramatically at the end of the study period when clinical testing rates declined in Davis, usage of at-home test kits increased, and TPR surpassed 5%. Mandatory asymptomatic testing continued for UC Davis through the end of the study period. In the absence of strong clinical testing programs, the relatively more stable WW thresholds. determined over this study period may serve as a future reference to assess relative SARS-CoV-2 infection dynamics for these sewersheds. We caution against the direct translation of the estimated WW thresholds to other sewersheds and analytical methods for WW. Further research is needed to investigate the application of this framework to other sewersheds, for alternative WW analytical methods, and other respiratory and enteric pathogens present in WW.

There are some limitations that are worth considering in our modeling framework. One of the limitations of this framework is that it requires access to both WW and test data for continued adaptation, which implies continuous community monitoring through testing. The capacity of current testing programs has decreased significantly with recent transitions to a new normal and the implementation of prevention and surveillance mechanisms such as vaccines, at-home tests, and WW surveillance. New limited testing may lead to passive case-finding (i.e., only those most likely to be infected are tested). The TPR may thus overestimate the current burden of the disease under these conditions. It is important to highlight that reduction in information poses new challenges and limitations in COVID-19 monitoring. With reductions in testing, public health authorities must decide between an indicator that overestimates the burden of the disease (TPR) and a projection that underestimates the burden of the disease (case counts). We propose to use the TPR in combination with WW measurements to reduce bias in the estimation of the burden of disease. To bypass the inherent limitations of TPR in estimating disease spread, one could consider hospitalizations. However, publicly available hospitalization is only available at the county level, and only one or maybe a few cities may have monitoring for WW, which might not represent the whole county.

In our study, the relationship between WW concentrations and TPR changed when TPR increased. This was likely due to changes in test-seeking behavior and test availability. Viral shedding may also change through time due to changes in vaccination status, acquired immunity, and changes in transmission patterns for different viral variants—although there is limited evidence available from fecal shedding studies. WW thresholds were estimated herein based on TPR for a community where testing rates were extraordinarily high, given the population size. While it is not feasible at this time to establish a priori public health thresholds based on WW concentrations alone to estimate the burden of the disease through time, a record of historical values and thresholds provides meaningful context to guide public health authorities as new waves of infection arise.

## MATERIALS AND METHODS

The City of Davis and UC Davis Campus WW collection areas (commonly referred to as sewersheds) are geographically adjacent. The analysis includes laboratory-confirmed incident COVID-19 cases and WW data from 1 July 2021, to 1 July 2022, for Davis and UC Davis. This period of study captured three waves of the pandemic, namely the Delta variant (dominant from 1 July 2021 to 14 December 2022), the Omicron variant BA.1 (dominant from 15 December 2021, to 15 March 2022), and Omicron variants BA.2, BA.3, BA.4, and BA.5 (dominant from 16 March 2022, to 1 July 2022); hereafter Omicron variants will be denoted as one BA.2–5 wave. These periods were defined according to the dominance of a variant as reported by the California Department of Public Health (34).

**Fig 5:**
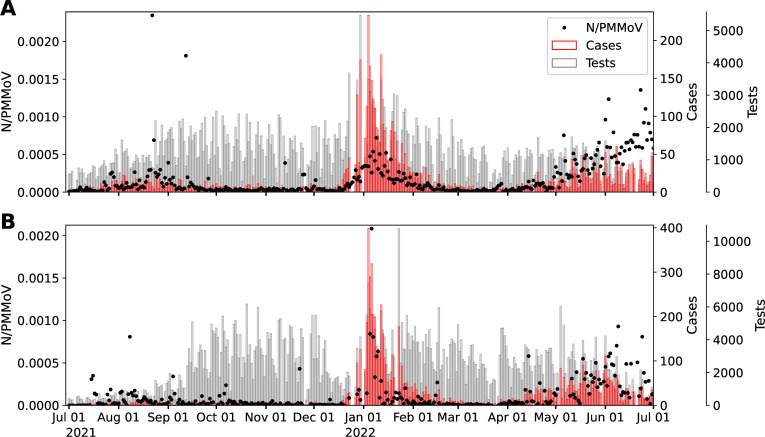
Normalized wastewater data (*N*/PMMoV), no. of COVID-19 tests conducted (Tests), and positive cases (Cases) for (**A**) Davis and (**B**) UC Davis.

**Fig 6:**
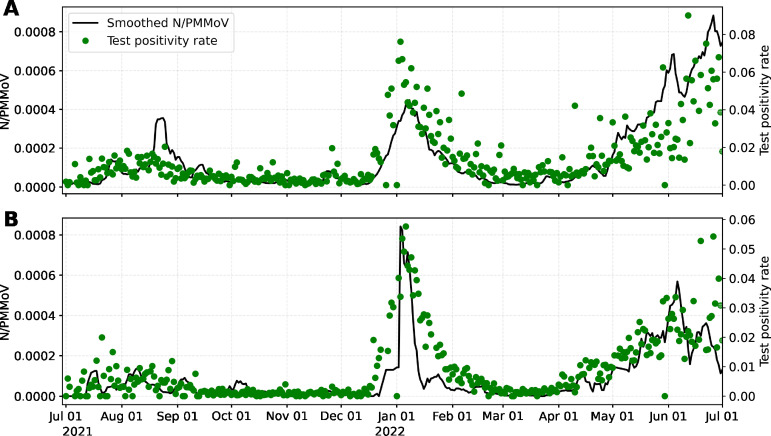
Seven days trimmed mean of wastewater data (Smoothed *N*/PMMoV) and daily TPR for (**A**) Davis and (**B**) UC Davis (1 July 2021–1 July 2022).

Daily COVID-19 cases and tests for Davis were provided by Healthy Davis Together (HDT) (35). HDT is the community pandemic response program launched in Davis from September 2020 to 30 June 2022, to mitigate the spread of COVID-19. HDT involved a broad set of interventions, including free saliva-based asymptomatic testing with high throughput methods to process large volumes of tests. Testing and cases for UC Davis come from the campus community COVID-19 screening program, which includes mandatory completion of biweekly asymptomatic tests to access campus facilities. The study site is thus unique compared with most WW surveillance regions in that there was an extraordinarily high number of clinical tests performed in both sewersheds during the study period (Fig. 5) for relatively small population size (425,314 tests were performed over the study period by Davis and 835,785 for UC Davis for approximately 66,799 residents in the combined surveillance regions). The high number of tests resulted in TPR ≤0.05 for the majority of the study period in both locations (Fig. 6). These conditions provide a useful context for estimating WW thresholds corresponding to the CDC-defined TPR thresholds for community transmission levels of SARS-CoV-2 (31). The WHO contends that disease dynamics based on case data can be confidently tracked when TPR ≤0.05 (21). Otherwise, when TPR ≥0.05, WW measurements offer a more robust measure of true disease dynamics (19).

### Wastewater settled solids methods

WW settled solids for the Davis wastewater treatment plant (WWTP) were collected daily from the primary clarifier, transported on the same day of collection to the analytical laboratory, and processed within 24 h as previously described (36). WW settled solids were obtained from daily composite influent samples from UC Davis WWTP. Composite influent samples were collected using a refrigerated autosampler (Hach Sigma 900 MAX) located at the WWTP headworks and programmed to collect flow-weighted influent sample volumes every 20 min for a total volume of 19 L in 24 h. Composite influent samples were then transferred to one or two 4 L low-density polyethylene containers (LDPE Cubitainers, Thermo Scientific I-Chem) and stored at 4°C prior to settling (up to 6 d of storage). Each 4 L sample was pasteurized in a 60°C water bath for 45 min immediately prior to settling. Pasteurized influent samples were inverted to mix, poured into a 3-gallon high-density polyethylene conical vessel equipped with a sampling port (FF3G, FastFerment), and left to settle for 2 h. Settled solids were obtained from either 4 L or 8 L of influent from a single day (two 4 L samples were combined into one settling vessel when 8 L was used). Settled solids were collected by dispensing from the bottom of the settling vessel into one or two 50 mL polypropylene centrifuge tubes (for 4 L or 8 L initial volume, respectively). If two tubes of settled solids were obtained, the supernatants were carefully decanted from each, and the remaining settled solids were combined. Between sampling episodes, the settling tanks were emptied, and the tank and sampling port valves were bleached (10% commercial bleach for 1 h), rinsed with deionized water, and left to air dry. Samples of settled solids were stored at 4°C and subsequently transported on ice in a cooler by a courier to the laboratory. Sample processing was completed within 8 d of initial sample collection. Sample RNA extraction, purification, and droplet digital reverse transcriptase PCR (ddRT-PCR) followed the same protocol as for the Davis samples. These protocols are described in detail elsewhere (37, 38).

We normalize the SARS-CoV-2 RNA concentration determined (*N* gene copies per gram dry weight solids) by the concentration of mild pepper mottle virus (PMMoV gene copies per gram dry weight solids) to yield the dimensionless metric, *N*/PMMoV. The *N* gene is present in all variants of the virus. PMMoV is a highly abundant RNA virus detected broadly in WW (39, 40). PMMoV serves as a process control such that normalization of *N* by PMMoV for each sample helps correct SARS-CoV-2 concentrations for virus extraction efficiency. PMMoV is also often used to account for variations in population size, rainfall, and water usage between different WW collection areas (41). We expect these latter factors to have less of an effect on *N* gene concentrations determined herein because water is removed from the WW settled solids samples prior to sample analysis and concentrations are reported in terms of the dry weight of the dewatered solids. Fig. 5 shows the normalized *N* gene concentration.

Given that WW signals are often noise corrupted, we applied a 7-d trimmed mean for daily WW data (smoothed *N*/PMMoV) to reduce uncertainty and minimize daily fluctuations. The smoothed data are later correlated to raw TPR (Fig. 6).

Table 2 illustrates Pearson’s correlation coefficient between the WW data (smoothed *N* gene and smoothed *N*/PMMoV) and clinical data (cases and TPR) in different waves. An improvement in correlation is observed when using TPR, compared with other cases. In general, an increase in correlation is also observed when using the normalized signal (*N*/PMMoV). Additionally, Table 2 highlights a notable change (from Omicron to BA.2–5 wave) in the correlation between WW and case data, while the correlation between WW data and TPR showed a relatively small change. These observations suggest that one of the primary factors responsible for the shift in the WW-to-cases relationship could be the reduction in the number of tests conducted during the second wave (see Fig. 5).

**TABLE 2:** Correlations between WW data (*N* gene or *N*/PMMoV) and daily TPR or COVID-19 positive cases (Cases) for Davis and UC Davis by wave

	Test positivity rate				Cases			
	All	Delta	Omicron	BA.2–5	All	Delta	Omicron	BA.2–5
**Davis**								
*N* gene	0.68	0.32	0.70	0.65	0.36	0.42	0.67	0.55
*N*/PMMoV	0.71	0.35	0.76	0.70	0.39	0.44	0.73	0.55
**UC Davis**								
*N* gene	0.76	0.22	0.74	0.71	0.64	0.01	0.65	0.51
*N*/PMMoV	0.77	0.35	0.79	0.71	0.62	0.03	0.70	0.47

### Statistical model

We model TPR as a beta distribution using WW viral loads as a covariate, assuming a Bayesian approach. We assume $Y_{i}$ as the TPR at day i , defined as the ratio of the number of new positive cases among the number of tests performed at day i . Beta regression is a good choice of model for continuous data with response variables expressed as proportions.

The beta distribution is reparametrized as $Y_{i}∼ℬ\,\left(\mu_{i},\phi \right)$ , with mean $\mu_{i}$ and variance $\sigma_{i}^{2}=\mu_{i}\,\left(1-\mu_{i}\right)/\left(1+\phi \right)$ , ϕ is known as the precision parameter since, for fixed $\mu_{i}$ , the larger the ϕ value, the smaller the variance of $Y_{i};\phi^{-1}$ as a dispersion parameter (42). The mean $\mu_{i}$ can be expressed as a function of the linear predictor $\eta_{i}=\beta^{T}\,x_{i}$ , where $\beta ={\left(\beta_{0},\beta_{1},\ldots ,\beta_{p}\right)}^{T}$ is a (p+1) -dimensional vector of unknown regression coefficients (including the intercept), and $x_{i}={\left(1,x_{1},\ldots ,x_{p}\right)}^{T}$ is the vector of covariates plus a one for the intercept. In this study, only the 7 d trimmed mean of the WW data (smoothed *N*/PMMoV), denoted as $C_{i}$ , is included in the linear predictor ( p=1 ). Thus, the linear predictor is given by $\eta_{i}=\beta_{0}+\beta_{1}\,C_{i}$ , and the logit link, the inverse of the logistic function, is used in the beta regression (i.e., $\;logit(\mu_{i})=\log\left(\frac{\mu_{i}}{1-\mu_{i}}\right)=\eta_{i}$ ).

The aim of this inference problem is to estimate $\theta =\left(\beta_{0},\beta_{1}\right)$ from measurements of WW data, $C=(C_{1},C_{2},\ldots ,C_{n})$ , and COVID-19 TPR, $Y=(y_{1},y_{2},\ldots ,y_{n})$ . Thus, the likelihood function for the previous model is given by:


$$L(\theta |C,Y)=\prod_{i=1}^{n}\frac{1}{B(a_{i},b_{i})}y_{i}^{a_{i}-1}(1-y_{i}{)}^{b_{i}-1},$$

where $a_{i}=\mu_{i}\,\phi$ , $b_{i}=\phi -a_{i}$ , $\mu_{i}=1/(1+\exp\left(-\eta_{i}\right)$ is the mean (logistic function), $\eta_{i}=\beta_{0}+\beta_{1}\,C_{i}$ is the linear predictor, and ϕ is the dispersion parameter.

### Bayesian statistical approach

We adopt a Bayesian statistical approach, which is well suited to model multiple sources of uncertainty and allows for incorporating background knowledge of the model’s parameters. In this framework, a prior distribution, $\pi_{\Theta }\,\left(\theta \right)$ , is required to account for the unknown parameter θ in order to obtain the posterior distribution. Having specified the likelihood and the prior, we use Bayes’ rule to calculate the posterior distribution,


$$\pi_{\Theta |C,Y}(\theta |C,Y)=\frac{\pi_{\Theta }(\theta )L(\theta |C,Y)}{Z(Y)},$$

where $Z(Y)=\int \pi_{\Theta }(\theta )L(\theta |C,Y)d\theta$ is the normalization constant. The posterior distribution is simulated using an existing Markov chain Monte Carlo (MCMC) method, the t-walk algorithm (43).

### Bayesian sequential method

We adapt the sequential approach proposed in reference 3 to our model to update forecasts over time. The aim is to train the model using only a subset of the most recent data. The forecast is then updated sequentially in a sliding window of data.

**Fig 7:**
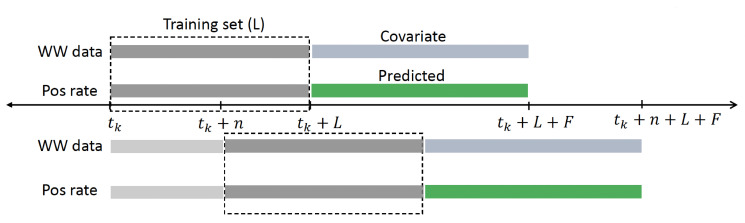
Schematic illustrating sequential modeling approach. The model is fitted with WW and TPR data from the training period (dark gray, denoted by L) starting at time *t*_*k*_. Then, the estimated parameters are used to predict the forecasting period (green, denoted by *F*). The training window is then moved *n* days forward. When new data become available, we update all forecasts; the latest posterior becomes the newest prior to the next training period.

We let L be the length in days of the period used to train the model. The data window is then moved forward every n day as new data become available. We set *t*_0_ as the first initial time to start the analysis and the subsequent initial times as $t_{k+1}=t_{k}+n$ . The training period is taken as $\left[t_{k},t_{k}+L\right]$ and the forecasting period as $\left[t_{k}+L,t_{k}+L+F\right]$ , see Fig. 7.

We denote $\theta_{k}=\left(\beta_{0}^{\left(k\right)},\beta_{1}^{\left(k\right)}\right)$ the model parameters to be inferred and the vectors of data as $C_{k,n}=(C_{k},\ldots ,C_{k+n}),Y_{k,n}=(y_{k},\ldots ,y_{k+n})$ at period k . Note that, from the beginning, $\theta_{k}$ is assumed to change in time within each forecast window. If k=0 , we postulate a prior distribution $\pi_{\Theta_{k}}\,\left(\theta_{k}\right)$ and a likelihood $L\,\left(\theta_{k}|x_{k,n},Y_{k,n}\right)$ previously described. The probabilistic prediction of *y*_*t*_, in the forecasting period $t\in \left[t_{k}+L,t_{k}+L+F\right]$ , is obtained by using the estimated parameters through the MCMC method and the WW data concentration ( $C_{k+L,F}$ ).

Afterward, the forecasting window is updated by setting $t_{k+1}=t_{k}+n$ , with n as the number of days until the next forecast. In the new training window $\left[t_{k+1},t_{k+1}+L\right]$ , we propose a new prior distribution $\pi_{\Theta_{k+1}}\,\left(\theta_{k+1}\right)$ for the model parameters $\theta_{k+1}$ using samples from the posterior distribution obtained in the previous forecast. Finally, we set k=k+1 and repeat the process described above to create a new forecast, see reference (3) for implementation details.

Regarding the elicitation of the parameters’ prior distribution for the first forecast, at k=0 , we assume a normal distribution for the parameters $\beta_{0}^{\left(k\right)}$ , $\beta_{1}^{\left(k\right)}$ , with mean and standard deviation (0,0) and (1,000, 1,000), respectively (i.e., noninformative priors). We set L to twice the length from symptoms onset to mild disease clinical outcome, namely 30 d, and F is chosen to be 10 d. The forecasting was updated every n=10 d.

### Action thresholds for WW concentration

We let Y be the TPR, and C corresponds to the SARS-CoV-2 RNA concentrations measured in WW. Then, the cumulative distribution function of Y given C is defined as $F_{Y|C}\,\left(y|c\right):=P\,\left(Y\leq y|C=c\right),$ which represents the probability that TPR is less than or equal to y , given that the SARS-CoV-2 RNA concentration in WW is c . Henceforth for simplicity, we will use F⁢(y|c) instead of $F_{Y|C}\,\left(y|c\right)$ without loss of generality. The quantile function $F^{-1}$ of Y given C=c is defined by


$$F_{c}^{-1}(p):=\inf\{y\in R:F(y|c)\geq p\},\;p\in (0,1).$$

The p -quantile of a data set is defined as the value where a p fraction of the data is below that value and (1-p) fraction of the data is above that value (e.g., the 0.5 quantile is the median).

Using the CDC thresholds for TPR values corresponding to low ( Y≤0.05 ), moderate ( Y∈(0.05,0.08) ), substantial ( Y∈(0.08,0.1) ), and high ( Y≥0.1 ) transmission levels of SARS-CoV-2 and the parameter estimates from the assumed beta regression model, we propose a methodology for estimating WW concentrations associated with TPR thresholds at a given point in time. We find the value of WW concentrations, c , such that with probability 1-α , the TPR is less than the CDC threshold y∈{0.5,0.8,0.1} (i.e., to find c such that $F_{c}^{-1}\,\left(\alpha \right)=y$ ). Note that α is the precision we set to estimate the threshold. With α=0.05 , we are being conservative and choose lower bounds of WW concentration values associated with TPR thresholds.

The proposed method for finding these thresholds, assuming that we have simulations of the posterior distribution for the parameter $\theta =\left(\beta_{0},\beta_{1}\right)$ , is given in Algorithm 1. First, we suggest a search grid for the WW viral load concentration. Then, for each concentration, we simulate the predicted posterior distribution of the TPR. Lastly, we calculate the α -quantile for each concentration level and find the concentration level that yields the quantile closest to the value of the desired TPR threshold.

Algorithm 1 **Finding threshold for WW using TPR.**

**Input:** A sample, $\theta_{1},\theta_{2},\ldots ,\theta_{N}$ , from the posterior distribution of Θ , where $\theta_{i}=(\beta_{0,i},\beta_{1,i}),\;i=1,\ldots ,N$ . The precision parameter ϕ , the threshold for the       TPR, $T_{p\,r}$ , and the probability α ;

**Output:** Threshold for WW concentration $T_{w\,w}$ ;

*Step 1*. Generate a grid of WW concentration, $c_{1},c_{2},\ldots ,c_{L}$ ;

**for** *l in 1* **to** *L* **do**

**  for** *i in 1* **to** *N* **do**

*   Step 2*. Simulate $Y_{l,i}∼B\,e\,t\,a\,\left(\mu_{l,i},\phi \right)$ , where $\mu_{l,i}=\frac{1}{1+\exp\left(-\eta_{l,i}\right)}$ and $\eta_{l,i}=\beta_{0,i}+\beta_{1,i}\,c_{l}$ ;

  Step 3. Compute the α -quantile of $Y_{l}=(Y_{l,1},\ldots ,Y_{l,N})$ , namely $Q_{l}$ ;

Step 4. Find $l^{*}$ such that $l^{*}=\underset{x}{\mathrm{argmin}}\;\left|Q_{l}-T_{p\,r}\right|$ ;

Step 5. Set $T_{w\,w}=c_{l^{*}}$

### Effective reproductive number

The number of people in a population who are susceptible to infection by an infected individual at any particular time is denoted by $R_{e}$ , the effective reproductive number. This dimensionless quantity is sensitive to time-dependent variation due to reductions in susceptible individuals, changes in population immunity, and other factors. $R_{e}$ can be estimated by the ratio of the number of new infections ( $I_{t}$ ) generated at time t , to the total infectious individuals at time t , given by $\sum_{s=1}^{t}I_{t-s}\,w_{s}$ , the sum of new infections up to time step t-1 , weighted by the infectivity function *w*_*s*_. We implement the method proposed in reference 44 to calculate the $R_{e}$ from the TPR estimated with the WW data.

Note that the TPR is an estimation of the proportion of infected persons. Therefore, if we multiply the TPR by a *T* value, representing the total number of tests carried out in the study period, we will have an estimate of the incidence, which we can use to compute the $R_{e}$ . Since $R_{e}$ is a scale-free metric, we should get similar results for different T values. We use T as the average of the tests carried out in Davis or UC Davis.

## Data Availability

The codes implemented for the study are available in the GitHub repository. Analyses were carried out using Python version 3.9.
